# A Multitask Network for the Diagnosis of Autoimmune Gastritis

**DOI:** 10.3390/jimaging11050154

**Published:** 2025-05-15

**Authors:** Yuqi Cao, Yining Zhao, Xinao Jin, Jiayuan Zhang, Gangzhi Zhang, Pingjie Huang, Guangxin Zhang, Yuehua Han

**Affiliations:** 1 State Key Laboratory of Industrial Control Technology, College of Control Science and Engineering, Zhejiang University, Hangzhou 310027, China; yuqicao@zju.edu.cn (Y.C.); 3190101004@zju.edu.cn (Y.Z.); 3200100354@zju.edu.cn (X.J.); 12432086@zju.edu.cn (J.Z.); huangpingjie@zju.edu.cn (P.H.); gxzhang@zju.edu.cn (G.Z.); 2 Department of Gastroenterology, The Second Affiliated Hospital, Zhejiang University School of Medicine, Hangzhou 310000, China; arbend@zju.edu.cn

**Keywords:** autoimmune gastritis, gastrointestinal endoscopy image, deep learning, multitask network, classification, image registration

## Abstract

Autoimmune gastritis (AIG) has a strong correlation with gastric neuroendocrine tumors (NETs) and gastric cancer, making its timely and accurate diagnosis crucial for tumor prevention. The endoscopic manifestations of AIG differ from those of gastritis caused by *Helicobacter pylori* (*H. pylori*) infection in terms of the affected gastric anatomical regions and the pathological characteristics observed in biopsy samples. Therefore, when diagnosing AIG based on endoscopic images, it is essential not only to distinguish between normal and atrophic gastric mucosa but also to accurately identify the anatomical region in which the atrophic mucosa is located. In this study, we propose a patient-based multitask gastroscopy image classification network that analyzes all images obtained during the endoscopic procedure. First, we employ the Scale-Invariant Feature Transform (SIFT) algorithm for image registration, generating an image similarity matrix. Next, we use a hierarchical clustering algorithm to group images based on this matrix. Finally, we apply the RepLKNet model, which utilizes large-kernel convolution, to each image group to perform two tasks: anatomical region classification and lesion recognition. Our method achieves an accuracy of 93.4 ± 0.5% (95% CI) and a precision of 92.6 ± 0.4% (95% CI) in the anatomical region classification task, which categorizes images into the fundus, body, and antrum. Additionally, it attains an accuracy of 90.2 ± 1.0% (95% CI) and a precision of 90.5 ± 0.8% (95% CI) in the lesion recognition task, which identifies the presence of gastric mucosal atrophic lesions in gastroscopy images. These results demonstrate that the proposed multitask patient-based gastroscopy image analysis method holds significant practical value for advancing computer-aided diagnosis systems for atrophic gastritis and enhancing the diagnostic accuracy and efficiency of AIG.

## 1. Introduction

Medical imaging plays a crucial role in capturing detailed internal structures of the human body and detecting disease-related abnormalities in a fast, efficient, and non-invasive manner, providing clinicians with intuitive and accurate diagnostic evidence. However, interpreting medical images often requires experienced physicians, and most images demand significant focus and time for thorough analysis before reaching a diagnostic conclusion. This not only places high demands on physicians but also increases their workload.

In recent years, the rapid advancement of digital image processing techniques, particularly deep learning algorithms, has significantly improved the ability of models to interpret medical image data [1,2,3]. With proper design and sufficient training, deep learning models have demonstrated superior speed and accuracy in image analysis, especially in medical applications. Consequently, an increasing number of researchers are exploring computer vision algorithms to develop computer-aided diagnosis (CAD) systems for medical image interpretation. These systems aim to enhance the efficiency and accuracy of medical image analysis, reduce physicians’ workload, improve diagnostic precision [4], and ultimately lower the cost of medical imaging examinations and diagnoses, making such tools more accessible to a broader population. A fundamental task in the development of CAD systems is medical image classification. This process involves identifying the anatomical structures depicted in the images and determining whether they contain disease-related lesions. The qualitative information derived from medical image classification assists physicians in forming preliminary diagnostic conclusions.

Currently, there are two main categories deep learning models for image classification: convolutional neural network (CNN) models, such as ResNet [5], DenseNet [6], and VGGNet [7]; and attention mechanism-based models, such as Vision Transformer [8] and Swin Transformer [9]. In the field of CADs, these classification models have been widely applied, and the main types of medical images oriented are pathological images, ultrasound images, X-ray images, endoscopic images, etc.

Autoimmune gastritis (AIG) is a subtype of chronic atrophic gastritis (CAG) and is often associated with symptoms such as achlorhydria, hypergastrinemia, and megaloblastic anemia. Over time, AIG can progress to conditions such as gastric neuroendocrine tumors (NETs) [10] and gastric cancer [11]. Initially, AIG was believed to have a higher incidence in Northern Europe, where pernicious anemia is prevalent. However, the widespread use of endoscopy has revealed a significant number of AIG cases in Asia, including China. Research on AIG diagnosis remains underdeveloped in these regions, and overall understanding of the disease is still insufficient. Endoscopic examination plays a critical role in diagnosing AIG, as physicians can identify potential signs of the disease through morphological changes in the gastric mucosa. These findings often prompt further diagnostic tests to confirm AIG. However, in regions with limited medical resources, physicians may be unfamiliar with AIG, leading to overlooked AIG-related features during endoscopic image interpretation and contributing to missed diagnoses. Endoscopically, AIG is characterized by atrophy in the gastric body and fundus, with little to no atrophy in the gastric antrum. This morphological feature, often referred to as “reverse atrophy”, contrasts with atrophic gastritis caused by *Helicobacter pylori* (*H. pylori*) infection, which primarily affects the gastric antrum and may extend to the gastric body. In AIG-related endoscopic image analysis, recognizing the pattern of gastric mucosal atrophy across different parts of the stomach is crucial for accurate diagnosis.

Here, we propose a patient-based method for analyzing endoscopic images. This multi-task approach simultaneously assesses gastric region information and mucosal atrophy status, providing valuable references to assist physicians in interpreting endoscopic findings. First, since some images captured during a single patient’s endoscopy may include overlapping gastric mucosal regions, analyzing each image independently without considering inter-image relationships can lead to information loss, ultimately affecting analysis accuracy. To address this, pairwise image registration is performed to establish relationships between images, which are then leveraged to optimize both anatomical region classification and lesion recognition. Next, to effectively capture the “reverse atrophy” feature associated with AIG, a patient’s images are classified into three gastric regions: fundus, body, and antrum. This classification provides spatial information about the anatomical regions depicted in the images. Simultaneously, lesion recognition is performed to determine whether gastric mucosal atrophy is present in the respective regions. Finally, the interpretability of the classification model is analyzed to address concerns about the opaque nature of deep learning predictions, thereby alleviating physicians’ hesitations regarding the trustworthiness of such models.

The experimental results demonstrate that the patient-based multitasking network proposed in this paper outperforms general image classification networks in both anatomical region classification and lesion recognition. Additionally, the method exhibits a certain degree of interpretability, making it more reliable for clinical applications.

The major contributions of this study are as follows:

- We propose a multitask deep learning network for classifying anatomical regions and recognizing lesions in gastroscopic images related to autoimmune gastritis, aiming to facilitate the automated diagnosis of this relatively rare disease;
- We design an image grouping method based on image registration, utilizing the Scale Invariant Feature Transform (SIFT) algorithm to analyze correlations among multiple gastroscopic images of a single patient. These correlations are used to construct a similarity matrix, which serves as the basis for grouping the patient’s gastroscopic images. By comprehensively analyzing all images within a group rather than focusing solely on a single image during classification, this approach effectively improves classification accuracy;
- We conduct an interpretability study on the proposed method to address concerns regarding the difficulty of interpreting the diagnostic outcomes of automated models. This effort aims to encourage the broader adoption and application of automated diagnostic methods for autoimmune gastritis.

## 2. Related Works

Currently, with the advancement of deep learning models, researchers have applied deep learning networks to medical image analysis. These efforts span various fields, including pathology images, X-ray images, CT images, ultrasound images, and endoscopic images [12,13,14,15,16,17,18,19]. Researchers have designed and developed medical image classification models tailored to the unique characteristics of each image type. The backbone network models used in these studies include CNNs, Transformers, and their variants.

In general, there are two main approaches for designing gastroscopy image processing models. One approach involves end-to-end disease diagnosis based on image classification, while the other focuses on extracting detailed information from images, such as the presence of polyps or bleeding. The latter approach, however, provides valuable information to assist physicians in their diagnostic process. For end-to-end disease diagnosis, Satoki Shichijo et al. utilized CNNs to diagnose gastritis caused by *H. pylori* infection, with the result of the experiment demonstrating that CNNs outperform physicians in terms of speed and accuracy for this diagnosis [20]. Additionally, Gong et al. developed a novel end-to-end CAD method by constructing a convolutional and relative self-attention parallel network (CRSAPNet), which was used to classify and diagnose AIG and gastritis caused by *H. pylori* infection, achieving high diagnostic accuracy [21]. For detailed information extraction, Zhao et al. proposed an adaptive cosine similarity network with a self-attention module (AdaSAN) for the automatic classification of gastrointestinal wireless capsule endoscopy images. Their method achieved excellent performance in classifying images of inflammation, bleeding, polyps, and normal tissues [22]. Rustam et al. combined MobileNet and CNN to propose a lightweight model called BIR for capsule gastroscopy (WCE) image classification, enabling efficient detection of bleeding lesions [23]. Furthermore, Mu et al. applied a deep convolutional neural network (DCNN) for the analysis of esophagogastroduodenoscopy images. Using ResNet-50 as the backbone of the classification model, their system conducted a layer-by-layer analysis of three-class gastroscopy images of gastritis, showing high specificity and accuracy in lesion classification [24].

In the context of multitask networks, recent advances have significantly boosted the performance and efficiency of medical image analysis by enabling joint optimization of related tasks such as segmentation and classification. Ling et al. proposed a one-stage multi-task attention network (MTANet), which efficiently classifies objects in medical images while generating a high-quality segmentation mask for each medical object [25]. Zhou et al. integrated segmentation and classification by utilizing multitask network to improve tumor characterization in 3D automated breast ultrasound (ABUS) images [26]. Moreover, Percannella et al. extended multitask learning to the pathology domain by jointly addressing intensity classification and specimen segmentation in HEp-2 cell images [27]. Collectively, these studies underscore that multi-task networks are not merely computationally efficient but are pivotal in capturing task synergies, reducing annotation costs, and advancing precision in medical image processing.

## 3. Method

### 3.1. Data Acquisition

The gastroscopic dataset used in this study was provided by the Department of Gastroenterology at the Second Affiliated Hospital of Zhejiang University School of Medicine. The dataset consists of 298 patients, and Table 1 presents the clinical information of these patients, including sex and age. These patients are diagnosed by experienced gastroenterologists with three conditions: AIG, *H. pylori* positive atrophic gastritis, and *H. pylori* negative chronic atrophic gastritis. AIG are diagnosed according to the Japanese diagnostic criteria of AIG [28]. *H. pylori* infection is determined by positive result on 13C-UBT and at least one of the two tests (histology and stool antigen test). CAG was diagnosed by gastroscopy manifestation and pathology result. The image resolutions varied, including sizes such as 1916 × 1076, 716 × 476, and 764 × 572. In this study, all images were uniformly resized to 512 × 512. The trial was approved by the hospital’s Ethics Committee, and written informed consent was obtained from all subjects.

**Figure 1:**
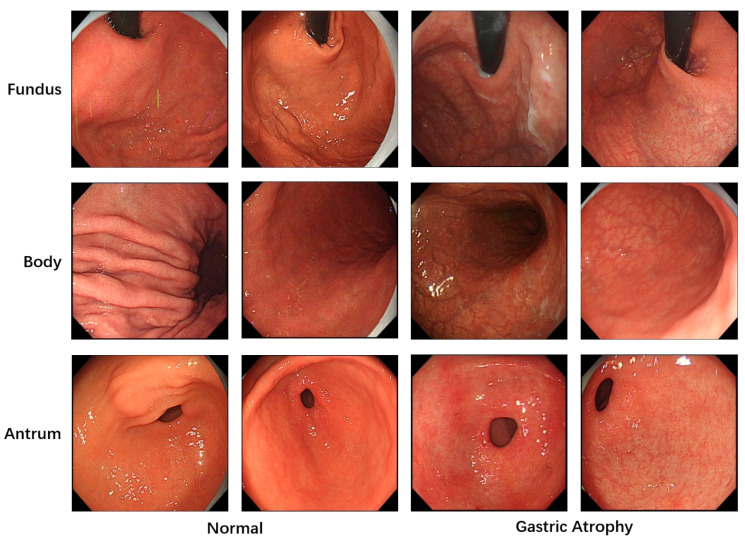
Examples of dataset images and their labels. Images in the left/right 2 columns: normal gastric mucosa/atrophic gastric mucosa. Images in the 1st/2nd/3rd row: fundus/body/antrum of the stomach.

All images used in this study were annotated by gastroenterologists to provide the necessary labels for model training. A total of 5981 images with image-level labels were utilized. As shown in Figure 1, these images were categorized into three classes based on gastric anatomical regions: fundus, body, and antrum. Regarding lesion classification, the images were divided into two categories: atrophic and normal gastric mucosa. The dataset was split into training, validation and test sets at an approximate ratio of 6:2:2. To prevent patient-level data leakage, images from the same patient were strictly assigned to either the training set or the validation set. Specifically, the training and validation sets consisted of 3529 and 1275 images from 201 and 49 patients, while the test set included 1177 images from 48 patients.

### 3.2. Overall Structure

**Figure 2:**
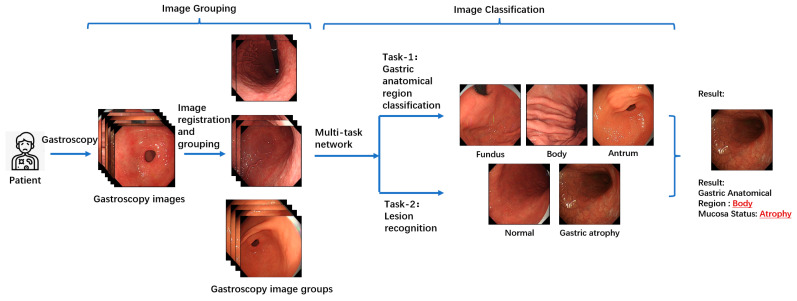
An overall schematic of the proposed method. A batch of gastroscopic images are acquired. Then, the image registration algorithm is used to calculate the similarity between the images in the batch to form similarity matrix, based on which, this batch of images are divided into groups. Finally, two classification tasks are carried out to extract the information related to autoimmune gastritis (AIG) in images, including the information about the anatomical region in the image and the information about the atrophic lesion of the gastric mucosa.

Figure 2 illustrates the overall workflow of the proposed multitask patient-based gastroscopy image analysis method. First, a batch of gastroscopic images is collected during a patient’s examination. In the image grouping step, an image registration algorithm is applied to calculate the similarity between images in the batch, forming a similarity matrix. Based on this matrix, the images are clustered into groups. Subsequently, two classification tasks—anatomical region classification and lesion recognition—are performed by the same network to extract information related to AIG, including the anatomical region depicted in each image and the presence of atrophic lesions in the gastric mucosa. By performing anatomical region classification, the corresponding gastric anatomical region (fundus, body, or antrum) of each endoscopic image is determined. Simultaneously, lesion classification enables assessment of mucosal atrophy severity. By integrating these two components of information, the atrophy status across all three gastric regions (fundus, body, and antrum) can be comprehensively evaluated. This integrated analysis allows for detection of characteristic patterns like “reverse atrophy”, thereby facilitating preliminary assessment of AIG likelihood.

### 3.3. Images Grouping

During gastroscopy, doctors often capture multiple images of the same gastric mucosal region. Since these images are taken sequentially, they tend to contain correlated regions. By combining information from these correlated regions for joint analysis, complementary information can be utilized, enabling the extraction of comprehensive features that represent the region. This, in turn, facilitates more effective classification. For instance, if Image A and Image B both depict the same gastric mucosal region, with minimal differences in position and morphology, it can be inferred that these two images provide redundant information about the gastric area and its associated pathology. Consequently, these images can complement each other, with the additional information supporting the classification of gastric regions and the recognition of lesions in gastroscopic images, thereby reducing the likelihood of model misclassification. Analyzing the interrelations among a patient’s images and leveraging the relationships between them offers greater clinical value than independently analyzing each image and performing simple information integration.

In the experiment, the classic image registration algorithm Scale Invariant Feature Transform (SIFT) [29] is applied. Being a well-known computer vision algorithm widely used for tasks such as image registration, object detection, and 3D reconstruction, SIFT is designed to extract key feature points and their descriptors from images that are invariant to scale and rotation, enabling precise image registration. The SIFT algorithm comprises four main stages: scale-space construction, key point detection, orientation assignment, and feature descriptor generation. First, the algorithm constructs a scale-space representation of the image using a Gaussian pyramid and computes the Difference of Gaussians (DoG) to detect potential key points, which typically correspond to prominent corners or edges in the image. Subsequently, key points are precisely localized at each scale level, with further refinement achieved by suppressing edge responses and low-contrast points, thereby enhancing the accuracy and robustness of key point detection. During the orientation assignment stage, SIFT assigns one or more dominant orientations to each key point based on the gradient direction distribution in the neighborhood of the key point, ensuring invariance to image rotation. Finally, in the feature descriptor generation stage, SIFT computes descriptors based on the histogram of gradients within the local neighborhood of each key point. These descriptors are normalized to improve robustness against changes in lighting and affine transformations. The matching of SIFT descriptors is typically performed using Euclidean distance or other similarity measures, and the random sample consensus method is employed to remove mismatched points, thereby achieving robust image registration.

For all gastroscopic images of a single patient, the SIFT algorithm is applied to compute pairwise registration results, obtaining the number of valid matching points between images. If the number of valid matching points between two images falls below a certain threshold, these images—despite sharing partially similar regions—are considered unrelated, and the similarity score of the image pair is set to 0.0. For image pairs exceeding the matching points threshold, the similarity between them is calculated based on the number of matching points, with a higher number of points indicating greater similarity. Using these pairwise similarity scores, a similarity matrix is constructed and subjected to hierarchical clustering, resulting in a series of image groups. These groups integrate highly similar images from all gastroscopic images of each patient. Within each group, models for anatomical region classification and lesion recognition are jointly applied. Since each group shares identical labels for region and lesion type, and a group of images collectively provides richer information than a single image, this approach leverages the shared information obtained through image registration for both classification tasks. As a result, this method effectively reduces the likelihood of mispredictions in both anatomical region classification and lesion recognition.

### 3.4. Anatomical Region Classification and Lesion Recognition

Given the specificity of AIG in the anatomical region of gastric mucosal atrophy, which is characterized by the so-called “reverse atrophy”, an accurate diagnosis of AIG requires a model capable of extracting key information for two distinct tasks. The first task involves identifying the gastric region represented in the image to pinpoint the specific area of the lesion. The second task involves assessing the condition of the gastric mucosa, specifically determining whether the image contains atrophic gastric mucosa. Based on these key features associated with AIG-related images, a multi-task network is employed to classify gastroscopic images. In the first task, automated anatomical region classification is performed on all gastroscopic images. Using existing labels, the images are categorized into three regions: fundus, body, and antrum, which serves as one of the prerequisites for diagnosing AIG. In the second task, a binary classification of lesion status is conducted on labeled images. The classification results identify the mucosal condition as either normal or atrophic. Specifically, if an image contains atrophic gastric mucosa, it is labeled and predicted as atrophic; otherwise, it is classified as normal gastric mucosa. By integrating the classification results of these two tasks, the atrophic status of the gastric mucosa across different regions of the stomach can be determined, enabling a preliminary assessment of the likelihood of AIG in the patient.

**Figure 3:**
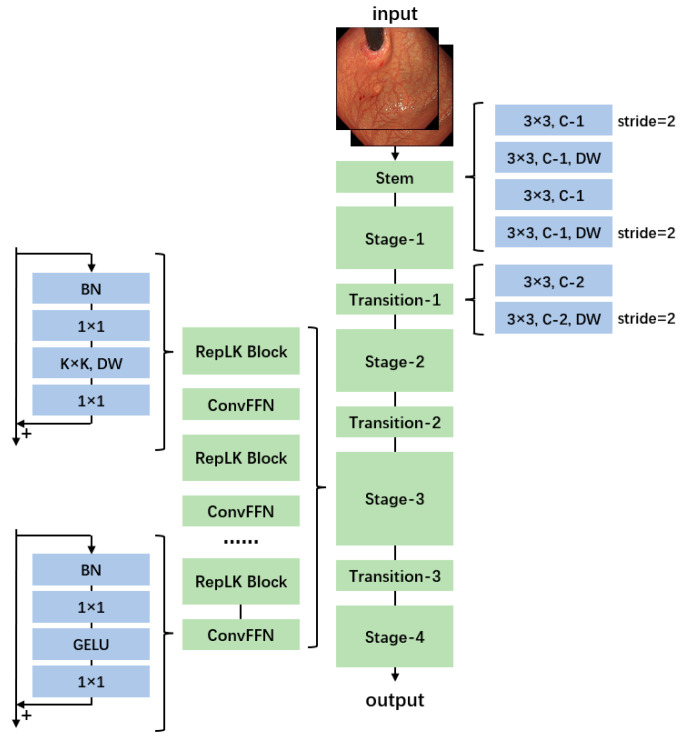
Structure of RepLKNet.

In this study, based on the fundamental characteristics of the dataset images and the classification objectives, RepLKNet [30], a convolutional neural network (CNN) architecture that utilizes large-kernel convolutions, is adopted as the backbone network for the two tasks. The structure of this model is shown in Figure 3. RepLKNet is an innovative CNN architecture that significantly expands the receptive field by introducing large-kernel convolutions while maintaining computational efficiency and effective parameter optimization. This approach overcomes the limitation of traditional convolutional networks, where the receptive field is often insufficient to capture global features due to the small size of convolutional kernels. Additionally, as a CNN-based model, RepLKNet avoids the lack of inductive bias observed in transformer-based models. The main idea of RepLKNet is to employ large convolutional kernels (e.g., 31 × 31 or larger) to cover a broader spatial range. However, directly introducing large-kernel convolutions would result in significantly increased computational costs and parameter complexity. To address this, structural re-parameterization techniques are used to efficiently compute large-kernel convolutions. During the training of RepLKNet, a composite module consisting of large-kernel decomposition and multi-branch structures is employed to substantially reduce computational burdens. In the inference phase, these branches are merged into an equivalent, more efficient single-branch model. This series of operations ensures that RepLKNet strikes a balance between a large receptive field and efficient training, making it well-suited for tasks like gastroscopic image analysis, where the dataset is relatively small, and both local and global information need to be effectively captured.

### 3.5. Metrics

The evaluation metrics include accuracy, mean precision, mean recall, and F1-score. Mean precision and mean recall, calculated as the averages across multiple classes, are used to evaluate the performance of the models. Considering that Transformer-based models require large-scale pretraining to learn image-related inductive biases, and to ensure the rigor of model performance comparisons, all models included in our experiments were initialized with pretrained weights from the ImageNet-1K dataset. Further training was then conducted using the internal dataset.

Deep learning models often face challenges related to interpretability due to their “black-box” nature, which makes it difficult to determine the basis for their final predictions. This characteristic poses a significant drawback in medical diagnosis, where strict and clear evidence is required. An unexplainable prediction process can lead to a lack of trust in the model’s outputs by physicians, thus diminishing its value as a reference for aiding diagnoses. To address this, the Grad-CAM [31] method is applied to visualize the regions of interest in the model during image classification, enhancing the interpretability of its predictions. Grad-CAM generates a heatmap by converting the output of a specific feature layer in the deep learning model into a visual representation. This highlights the areas the model focuses on when making predictions for a given image input, providing insights into the reasoning behind the model’s predictions. To better align with the final prediction results, the output of the last convolutional layer is selected as the target for Grad-CAM in this study.

## 4. Results

### 4.1. Results of Images Grouping

**Figure 4:**
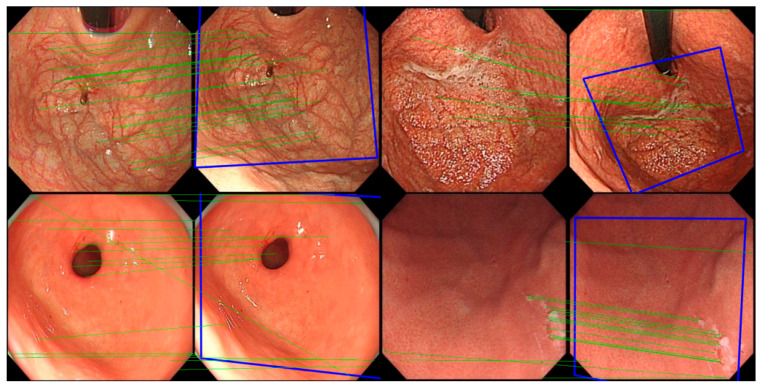
Examples of image registration (green lines are the feature point match lines, while blue boxes are the position of the left image in the right image, in order to make the presentation clearer, the number of match lines in the image was randomly reduced).

As shown in Figure 4, the registration algorithm successfully identifies a sufficient number of registration points between images that contain overlapping regions and are relatively similar, effectively aligning the images. Most of the identified registration points are accurately matched, with only a few mismatches observed. Furthermore, when there are enough registration points, the algorithm is able to preliminarily establish the relative positional relationship between the image pair.

**Figure 5:**
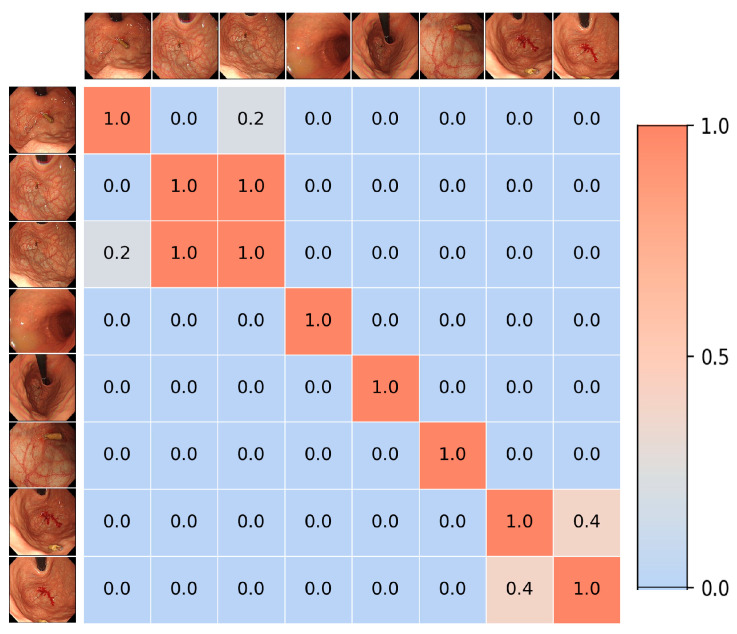
Example of the similarity matrix obtained in the experiment (for all image pairs with too few matches, the similarity was set to 0.0).

As shown in Figure 5, the similarity matrix derived from image registration indicates that image pairs with higher similarity are assigned larger similarity values. Based on this matrix, the first three images and the last two images are grouped into two clusters, while the remaining images are not included in any group. However, despite the strict constraints applied, the registration algorithm still fails to detect some similar regions between certain images. For example, the first and second images, as well as the sixth image and the first three images, are located in similar regions and share distinct markers, but the algorithm does not identify the relationships between them.

**Figure 6:**
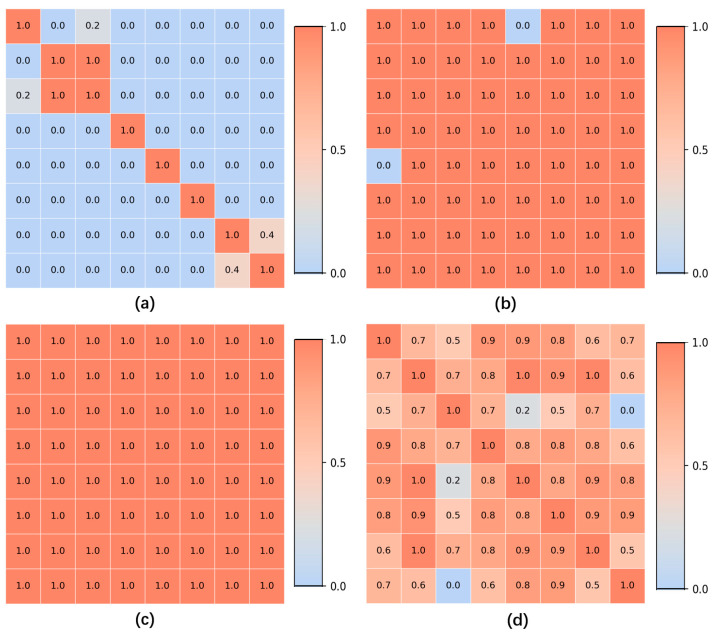
Examples of the similarity matrix obtained by different algorithms in the experiment. (**a**) The similarity matrix obtained through the SIFT algorithm. (**b**) The similarity matrix based on structural similarity (SSIM). (**c**) The similarity matrix based mean squared error (MSE). (**d**) The similarity matrix obtained based on histogram similarity.

As shown in Figure 6, traditional similarity algorithms, such as SSIM, may struggle with gastroscopic images, as these images often exhibit minimal differences in color and morphology, leading to generally high similarity scores across them. While traditional similarity computation methods can effectively match similar images, they may erroneously group entirely dissimilar images. This issue could result in a higher number of misclassified images during subsequent anatomical region classification and lesion recognition tasks, ultimately undermining the overall rigor of the classification approach. Therefore, traditional similarity algorithms are not employed in this research.

### 4.2. Results of Anatomical Region Classification

The results of anatomical region classification, as shown in Table 2, demonstrate that the RepLKNet model, which incorporates large-kernel convolution, outperforms all other models across various metrics. Furthermore, by combining RepLKNet with image grouping, our method achieves the best results, achieving an accuracy of 0.934 ± 0.005 (95% CI) and a precision of 0.926 ± 0.004 (95% CI). Anatomical region classification in gastroscopic images is particularly challenging due to significant variability in factors such as image angles, brightness, and other conditions. This requires models to capture global contextual information to accurately identify specific regions. While Transformer-based vision models, such as ViT, offer a strong global perspective, their lack of inherent inductive bias results in a higher dependency on large-scale training data. In this study, the dataset was not suitable for pretraining, which significantly limited the performance of models like ViT.

**Figure 7:**
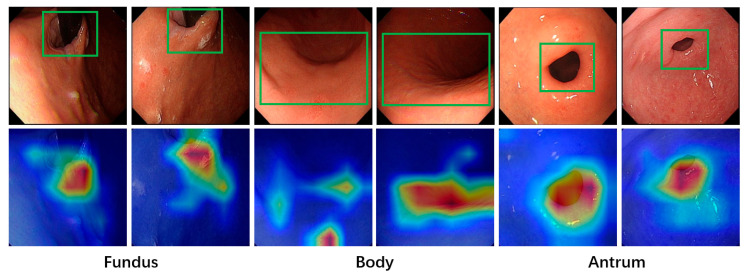
Visualization of anatomical region classification. The regions marked by green bounding boxes correspond to characteristic feature areas of their respective anatomical sites, demonstrating the model’s ability to identify clinically relevant patterns.

As shown in Figure 7, the Grad-CAM visualization results demonstrate that during anatomical region classification, the model concentrates on the most distinct feature regions within the relevant gastric parts. These feature regions differ across the various areas of the stomach. For the fundus, the key features include the fundal vault, the cardia, and the gastroscope tube extending from it, which the model successfully identifies. In the case of the antrum, the most prominent features are the pyloric duct and pylorus, which the model also detects effectively. For the body, the regions of focus generally cover a larger area compared to the other two regions. This is because the body lacks highly localized features like the pylorus or cardia, requiring the model to adopt a more holistic perspective to accurately analyze the image and identify its characteristics.

### 4.3. Results of Lesion Recognition

As shown in Table 3, among the backbone networks used for lesion recognition, RepLKNet, which leverages large-kernel convolutions, demonstrated the best performance. Additionally, by incorporating extra information obtained through image grouping, our method outperforms other models in this task, achieving an accuracy of 0.902 ± 0.010 (95% CI) and a precision of 0.905 ± 0.008 (95% CI). Lesion recognition, particularly in detecting atrophic gastric mucosa, can be especially challenging due to the subtle differences between atrophic and surrounding normal mucosa. To effectively identify atrophic lesions, the model requires a broader receptive field to simultaneously analyze both normal and atrophic mucosa within a single receptive area. This capability allows the model to directly compare mucosal conditions, which is crucial for detecting smaller or milder atrophic regions. These characteristics make RepLKNet particularly well-suited for this task, as it excels at capturing global context while maintaining the efficiency and precision needed to distinguish subtle variations in gastric mucosa.

**Figure 8:**
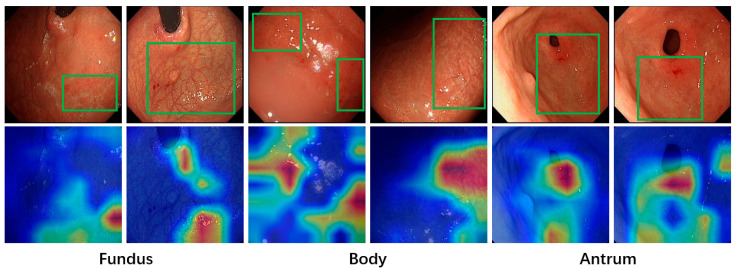
Visualization of lesion recognition. Areas demarcated by green bounding boxes represent regions with gastric mucosal atrophy.

As illustrated in Figure 8, the model performs well in identifying regions with distinct gastric mucosal features for lesion classification. The distribution of heatmaps reveals that the brightness of gastroscopic images significantly influences the model’s ability to accurately recognize lesion areas. The model tends to focus on brighter regions, which often highlight mucosal details more prominently. This observation suggests that the brightness and contrast of gastroscopic images play a crucial role in guiding the model’s attention and enhancing its ability to detect atrophic mucosa. It emphasizes the importance of preprocessing and standardizing image brightness to improve model performance and ensure consistent identification of lesion areas.

## 5. Discussions

This study employs a method based on image registration to group gastroscopic images, effectively leveraging the correlations among images from the same patient. Combined with the RepLKNet model based on large-kernel convolutions, the method efficiently extracts anatomical regions and lesion information related to AIG in gastroscopic images. The study contributes to the automated auxiliary diagnosis of AIG and demonstrates clear advantages over both traditional and other automated methods. Conventional diagnosis of AIG requires physicians to manually evaluate endoscopic images to determine these features—a time-consuming process subject to inter-observer variability, particularly in lesion-anatomy correlations. The proposed automated deep learning system mitigates these limitations by offering standardized, efficient image interpretation. Compared to studies focusing solely on lesion recognition [36], this method incorporates anatomical region information and explicitly links lesion analysis with AIG diagnosis, better aligning with clinical diagnostic needs. Furthermore, unlike approaches that analyze single endoscopic images in isolation, our system comprehensively evaluates multiple images from each patient to identify inter-image correlations, thereby optimizing both anatomical region classification and lesion recognition. In contrast to existing automated AIG diagnostic research [37], the methodology proposed in this study places greater emphasis on analyzing interpretable, specific pathological features that are clinically meaningful for AIG identification.

In practice, this method can be integrated into the gastroscopy workflow in two ways. The first approach is embedding it into gastroscopy devices to provide real-time alerts on relevant lesions and predict the probability of AIG. The second approach is incorporating it into telemedicine services to assist regions with limited medical resources. By analyzing gastroscopy images while ensuring patient privacy, this method provides clinically useful decision support for AIG diagnosis, thereby reducing the risk of missed diagnoses due to disparities in medical expertise.

This research has two limitations. First, the current dataset size remains limited and requires further expansion to improve the model’s performance and generalizability. Second, the data were collected from a single institution, and obtaining standardized multi-center data remains challenging. This limitation hinders validation of the model’s performance across different institutional datasets, consequently restricting its practical application in diverse clinical settings. In the future, to continue expanding the internal dataset and enhance model performance, we will systematically collect patient data from multiple physicians. Additionally, we will pursue partnerships with other hospitals and research centers. This multicenter approach will allow us to gather comprehensive AIG-related patient data, thereby advancing automated diagnosis research. Additionally, we aim to explore automated analysis methods for other AIG-related indicators, such as pathology and serological testing, with the ultimate goal of achieving autonomous AIG diagnosis based on multimodal information. Building upon this diagnostic model, we intend to integrate it with telemedicine platforms, enabling online autonomous image interpretation. This integration would allow even medically underserved regions to benefit from an online AIG diagnostic system, improving awareness and screening of AIG while reducing the likelihood of missed diagnoses.

## 6. Conclusions

In this study, we propose a patient-based multi-task analysis method for gastroscopic images that integrates the relational information across all images from a single patient. Using a unified network, the approach classifies both the gastric regions captured in the images and the presence or absence of mucosal atrophy. To extract inter-image relationships within a patient’s gastroscopic data, an image registration algorithm is applied to identify correlations and construct a similarity matrix. This similarity matrix is then used for image grouping. Then, a large-kernel convolution-based deep learning network, RepLKNet, is used to perform comprehensive classifications of gastric regions and lesions for the grouped images.

Experimental results show that grouping multiple gastroscopic images from the same patient and performing joint analysis significantly improves the accuracy of anatomical site and lesion recognition. The use of RepLKNet, a model based on large-kernel convolutions, strikes a balance between receptive field size and local inductive bias, demonstrating superior performance in gastroscopic image analysis tasks with limited data. The method demonstrated high accuracy in both gastric anatomical region classification (93.5%) and lesion recognition (90.7%). In summary, by leveraging the intrinsic characteristics of gastroscopic image data, the proposed method achieves superior performance in gastric anatomical region and lesion recognition, thereby facilitating a unified analysis framework specifically optimized for the automated auxiliary diagnosis of AIG.

## Figures and Tables

**Table 1:** Clinical information of patients.

	AIG	*H. pylori* Positive	*H. pylori* Negative
Number	96	102	100
Sex (men/women)	45/51	51/51	52/48
^1^ Age (years)	62.1 ± 8.1 (41, 71)	58.8 ± 7.5 (45, 65)	58.5 ± 11.3 (32, 79)

^1^ Indicates mean ± std, and (min, max).

**Table 2:** Results of anatomical region classification.

Models	Accuracy	Precision	Recall	F1-Score
ResNet-50 [5]	0.875	0.864	0.853	0.858
ResNeXt-50 [32]	0.897	0.883	0.886	0.884
DenseNet-121 [6]	0.891	0.893	0.890	0.890
SEResNet-50 [33]	0.893	0.876	0.881	0.878
PoolFormer-s24 [34]	0.891	0.880	0.872	0.875
SwinTransformer [9]	0.910	0.908	0.889	0.898
VisionTransformer [8]	0.888	0.872	0.873	0.873
ConFormer [35]	0.883	0.884	0.884	0.883
RepLKNet-31B [30]	0.920	0.914	0.921	0.918
**Ours**	**0.93** **4**	**0.92** **6**	**0.93** **7**	**0.93** **2**

**Table 3:** Results of lesion recognition.

Models	Accuracy	Precision	Recall	F1-Score
ResNet-50 [5]	0.833	0.833	0.833	0.833
ResNeXt-50 [32]	0.847	0.853	0.829	0.837
DenseNet-121 [6]	0.854	0.854	0.854	0.854
SEResNet-50 [33]	0.870	0.873	0.868	0.870
PoolFormer-s24 [34]	0.866	0.865	0.866	0.866
SwinTransformer [9]	0.873	0.872	0.874	0.872
VisionTransformer [8]	0.837	0.837	0.838	0.837
ConFormer [35]	0.869	0.869	0.870	0.869
RepLKNet-31B [30]	0.882	0.883	0.874	0.879
**Ours**	**0.90** **2**	**0.90** **5**	**0.** **895**	**0.90** **0**

## Data Availability

The data cannot be made publicly available upon publication because they contain sensitive personal information.

## References

[1] Pozza A., Zanella L., Castaldi B., Di Salvo G. (2024). How will artificial intelligence shape the future of decision-making in congenital heart disease?. J. Clin. Med..

[2] Mahmood T., Rehman A., Saba T., Nadeem L., Bahaj S.A.O. (2023). Recent advancements and future prospects in active deep learning for medical image segmentation and classification. IEEE Access.

[3] Li X., Zhang L., Yang J., Teng F. (2024). Role of artificial intelligence in medical image analysis: A review of current trends and future directions. J. Med. Biol. Eng..

[4] Wenderott K., Krups J., Zaruchas F., Weigl M. (2024). Effects of artificial intelligence implementation on efficiency in medical imaging—A systematic literature review and meta-analysis. NPJ Digit. Med..

[5] He K., Zhang X., Ren S., Sun J. Deep residual learning for image recognition. Proceedings of the IEEE Conference on Computer Vision and Pattern Recognition.

[6] Huang G., Liu Z., Van Der Maaten L., Weinberger K.Q. Densely connected convolutional networks. Proceedings of the IEEE Conference on Computer Vision and Pattern Recognition.

[7] Simonyan K., Zisserman A. (2014). Very deep convolutional networks for large-scale image recognition. arXiv.

[8] Dosovitskiy A. (2020). An image is worth 16 × 16 words: Transformers for image recognition at scale. arXiv.

[9] Liu Z., Lin Y., Cao Y., Hu H., Wei Y., Zhang Z., Lin S., Guo B. Swin transformer: Hierarchical vision transformer using shifted windows. Proceedings of the IEEE/CVF International Conference on Computer Vision.

[10] Massironi S., Gallo C., Elvevi A., Stegagnini M., Coltro L.A., Invernizzi P. (2023). Incidence and prevalence of gastric neuroendocrine tumors in patients with chronic atrophic autoimmune gastritis. World J. Gastrointest. Oncol..

[11] Lenti M.V., Rugge M., Lahner E., Miceli E., Toh B.H., Genta R.M., Block C.D., Hershko C., Di Sabatino A. (2020). Autoimmune gastritis. Nat. Rev. Dis. Primers.

[12] Zheng Y., Gindra R.H., Green E.J., Burks E.J., Betke M., Beane J.E., Kolachalama V.B. (2022). A graph-transformer for whole slide image classification. IEEE Trans. Med. Imaging.

[13] Li H., Chen Y., Chen Y., Yu R., Yang W., Wang L., Ding B., Han Y. Generalizable Whole Slide Image Classification with Fine-Grained Visual-Semantic Interaction. Proceedings of the IEEE/CVF Conference on Computer Vision and Pattern Recognition.

[14] Liu F., Tian Y., Chen Y., Liu Y., Belagiannis V., Carneiro G. Acpl: Anti-curriculum pseudo-labelling for semi-supervised medical image classification. Proceedings of the IEEE/CVF Conference on Computer Vision and Pattern Recognition.

[15] Dandıl E. (2018). A Computer-Aided Pipeline for Automatic Lung Cancer Classification on Computed Tomography Scans. J. Healthc. Eng..

[16] Fan Z., Gong P., Tang S., Lee C.U., Zhang X., Song P., Chen S., Li H. (2023). Joint localization and classification of breast masses on ultrasound images using an auxiliary attention-based framework. Med. Image Anal..

[17] Jin Y., Lu H., Zhu W., Huo W. (2023). Deep learning based classification of multi-label chest X-ray images via dual-weighted metric loss. Comput. Biol. Med..

[18] Huo X., Sun G., Tian S., Wang Y., Yu L., Long J., Zhang W., Li A. (2024). HiFuse: Hierarchical multi-scale feature fusion network for medical image classification. Biomed. Signal Process. Control..

[19] Togo R., Yamamichi N., Mabe K., Takahashi Y., Takeuchi C., Kato M., Sakamoto N., Ishihara K., Ogawa T., Haseyama M. (2019). Detection of gastritis by a deep convolutional neural network from double-contrast upper gastrointestinal barium X-ray radiography. J. Gastroenterol..

[20] Shichijo S., Nomura S., Aoyama K., Nishikawa Y., Miura M., Shinagawa T., Takiyama H., Tanimoto T., Ishihara S., Matsuo K. (2017). Application of convolutional neural networks in the diagnosis of Helicobacter pylori infection based on endoscopic images. EBioMedicine.

[21] Gong D., Yan L., Gu B., Zhang R., Mao X., He S. (2023). A Computer-Assisted Diagnosis System for the Detection of Chronic Gastritis in Endoscopic Images Using a Novel Convolution and Relative Self-Attention Parallel Network. IEEE Access.

[22] Zhao Q., Yang W., Liao Q. (2021). AdaSAN: Adaptive cosine similarity self-attention network for gastrointestinal endoscopy image classification. Proceedings of the 2021 IEEE 18th International Symposium on Biomedical Imaging (ISBI).

[23] Rustam F., Siddique M.A., Siddiqui H.U.R., Ullah S., Mehmood A., Ashraf I., Choi G.S. (2021). Wireless capsule endoscopy bleeding images classification using CNN based model. IEEE Access.

[24] Mu G., Zhu Y., Niu Z., Li H., Wu L., Wang J., Luo R., Hu X., Li Y., Zhang J. (2021). Expert-level classification of gastritis by endoscopy using deep learning: A multicenter diagnostic trial. Endosc. Int. Open.

[25] Ling Y., Wang Y., Dai W., Yu J., Liang P., Kong D. (2023). Mtanet: Multi-task attention network for automatic medical image segmentation and classification. IEEE Trans. Med. Imaging.

[26] Zhou Y., Chen H., Li Y., Liu Q., Xu X., Wang S., Yap P.T., Shen D. (2021). Multi-task learning for segmentation and classification of tumors in 3D automated breast ultrasound images. Med. Image Anal..

[27] Percannella G., Petruzzello U., Ritrovato P., Rundo L., Tortorella F., Vento M. Joint intensity classification and specimen segmentation on HEp-2 images: A deep learning approach. Proceedings of the 2022 26th International Conference on Pattern Recognition (ICPR).

[28] Kamada T., Watanabe H., Furuta T., Terao S., Maruyama Y., Kawachi H., Kushima R., Chiba T., Haruma K. (2023). Diagnostic criteria and endoscopic and histological findings of autoimmune gastritis in Japan. J. Gastroenterol..

[29] Lindeberg T. (2012). Scale Invariant Feature Transform. Scholarpedia.

[30] Ding X., Zhang X., Han J., Ding G. Scaling up your kernels to 31 × 31: Revisiting large kernel design in cnns. Proceedings of the IEEE/CVF Conference on Computer Vision and Pattern Recognition.

[31] Selvaraju R.R., Cogswell M., Das A., Vedantam R., Parikh D., Batra D. Grad-cam: Visual explanations from deep networks via gradient-based localization. Proceedings of the IEEE International Conference on Computer Vision.

[32] Xie S., Girshick R., Dollár P., Tu Z., He K. Aggregated residual transformations for deep neural networks. Proceedings of the IEEE Conference on Computer Vision and Pattern Recognition.

[33] Hu J., Shen L., Sun G. Squeeze-and-excitation networks. Proceedings of the IEEE Conference on Computer Vision and Pattern Recognition.

[34] Yu W., Luo M., Zhou P., Si C., Zhou Y., Wang X., Feng J., Yan S. Metaformer is actually what you need for vision. Proceedings of the IEEE/CVF Conference on Computer Vision and Pattern Recognition.

[35] Gulati A., Qin J., Chiu C.C., Parmar N., Zhang Y., Yu J., Han W., Wang S., Zhang Z., Wu Y. (2020). Conformer: Convolution-augmented transformer for speech recognition. arXiv.

[36] Yang J., Ou Y., Chen Z., Liao J., Sun W., Luo Y., Luo C. (2022). A benchmark dataset of endoscopic images and novel deep learning method to detect intestinal metaplasia and gastritis atrophy. IEEE J. Biomed. Health Inform..

[37] Chen S., Xu L., Yan L., Zhang J., Zhou X., Wang J., Yan T., Wang J., He X., Ma H. (2025). A novel endoscopic artificial intelligence system to assist in the diagnosis of autoimmune gastritis: A multicenter study. Endoscopy.

